# Author's reply

**DOI:** 10.4103/0974-7788.64404

**Published:** 2010

**Authors:** Suzan Sanavi, Reza Afshar

**Affiliations:** *Internal Medicine Department, Shahed University, Mustafa Khomeini Hospital, Tehran, Iran E-mail: s2sanavi@yahoo.com*

Sir,

The presented case in our article had consumed ginger by herself without consulting a herbalist. Because of the safety of herbal preparations and habitual consumption of them, many people in Iran consume these preparations without any knowledge about their adverse effects. It is important to note that even though the patient had taken small doses of ginger for the second time, she had experienced similar symptoms. This reveals that the patient might have had a sensitivity reaction to ginger even in small doses.

